# Cross-sectional analysis of physical activity in 2–4-year-olds in England with paediatric quality of life and family expenditure on physical activity

**DOI:** 10.1186/s12889-019-7129-y

**Published:** 2019-06-28

**Authors:** Laura Tinner, Ruth Kipping, James White, Russell Jago, Chris Metcalfe, William Hollingworth

**Affiliations:** 1 Population Health Sciences, Bristol Medical School, University of Bristol, Canygne Hall, Bristol, BS8 2BN UK; 2 Centre for Trials Research, Cardiff University, Cardiff, CF14 4YS UK; 3 Centre for Exercise, Nutrition & Health Sciences, School for Policy Studies, University of Bristol, Bristol, BS8 1TZ UK; 4 The National Institute for Health Research Collaboration for Leadership in Applied Health Research and Care West (NIHR CLAHRC West) at University Hospitals Bristol NHS Foundation Trust, Bristol, UK

**Keywords:** Physical activity, Preschool children, Quality of life, PedsQL, Expenditure, MVPA

## Abstract

**Background:**

Many children do not meet the recommended level of daily physical activity, even with the widely acknowledged health benefits associated with being physically active. There is a need to establish factors related to physical activity in children so that public health interventions may be appropriately designed. We investigated the association between Pediatric Quality of Life Inventory (PedsQL), family expenditure on physical activity and objectively measured daily physical activity in 2–4-year-old children.

**Methods:**

Cross-sectional study with a sample of 81 UK preschool children taking part in the NAPSACC UK feasibility randomized controlled trial. Descriptive statistics are presented. We undertook Student t-tests to establish differences in physical activity by gender, age, parental education and nursery versus non-nursery days. Mixed effects linear regressions were used to model the association between minutes spent physically activity, minutes spent in moderate-to-vigorous (MVPA) physical activity and PedsQL scores (physical and psychosocial) and family expenditure on physical activity.

**Results:**

Most children (88.9%) did not engage in the recommended 180 min daily physical activity. There was mean (SD) of 141.9 (33.1) daily minutes of physically activity and 22.2 min per day (SD = 9.9) of MVPA. Boys and older children were more physically active. Children were more active on nursery days. There was no difference in physical activity by parental education. Half of the sample parents (50.6%) spent less than £9.00 weekly on their pre-schooler’s physical activity. Children within the highest tertile of PedsQL physical functioning scores had higher levels of MVPA (3.6, 95% CI: − 1.3–8.4, *p*-value 0.15), although confidence intervals crossed the null in the adjusted model. We found no evidence of an association between positive PedsQL psychosocial scores, or higher parental expenditure on physical activity, with the physical activity variables.

**Conclusions:**

Children in this sample were not meeting the recommended 180 min of daily physical activity. The 2–4-year-olds were most active on nursery days. There is no evidence of an association between better PedsQL physical scores and higher levels of MVPA. There was no evidence of an association between expenditure on physical activity and time spent physically active. Further examination in larger representative datasets is needed.

**Electronic supplementary material:**

The online version of this article (10.1186/s12889-019-7129-y) contains supplementary material, which is available to authorized users.

## Background

Research has highlighted the importance of physical activity in young children, indicating benefits related to physical and psychological health [1]. The UK guidelines for children under 5 years state that children should engage in at least 180 min of physical activity per day, which includes light physical activity (LPA) and moderate-to-vigorous (MVPA). In 2011, only 10% of children under 5 met this recommendation and those aged 3–4 years were sedentary for on average 10–11 h per day [2].

There are no specific guidelines in the UK regarding the amount of MVPA under 5-year olds should engage in. The UK physical activity guidelines are currently being reviewed and will be published in 2019 by the four country Chief Medical Officers, which is expected to be based upon the recommendation that pre-schoolers should progress to 60 mins of daily MVPA by age 5 [3]. The guidelines for children between 6 and 17 years old in England recommends 60 min in MVPA per day [4], however, a birth cohort study in the UK found only 51% of primary school children met this recommendation [5]. Establishing behaviours in preschool children may result in increased levels of physical activity in later life, as physical activity behaviours track from childhood into adulthood [6]. There is limited and varied evidence of pre-schoolers levels of MVPA, with most of the evidence not gathered in the UK. A study of physical activity in children aged 3–5 years in the USA found that in childcare, children only spend 3% of time in MVPA [7]. Whereas a cross-sectional study in Southampton, England found that while 3–4-year-olds met the UK physical activity guidelines, the children spent the majority of their time in LPA [8]. There is therefore a need to increase physical activity and MVPA into the daily routines of pre-school children [9].

Childcare usage is high in the UK, with national statistics in 2018 finding that 72% of 2-year-olds and 94% of 3–4-year-olds benefited from universal funded early education places [10]. Therefore, nurseries remain an important setting for research on physical activity amongst young children.

A number of qualitative studies have highlighted the potential barriers to children and adolescents being physically active as lack of financial support from parents and lack of transportation [11–14]. Differential access to formal and paid for physical activities such as swimming is also a suggested contributor to inequalities in childhood obesity prevalence [11, 15, 16]. It is therefore important to investigate whether family expenditure on a child being physically active is associated with physical activity in children. However, to our knowledge, no study has measured this association in pre-school age children.

Health-related quality of life (HRQOL) is a multidimensional construct that includes physical, emotional and social health dimensions as delineated by the WHO [17, 18]. In children, HRQOL is an important indicator of everyday functioning [19] so there is a rationale that HRQOL may be associated with physical activity [20]. It is important to examine this association, as health promotion strategies could have a dual benefit in improving both physical activity levels and quality of life in children [21].

There is inconsistent evidence on the relationship between physical activity and HRQOL in children. One cross-sectional study found an association between HRQOL and physical activity in children and adolescents, with less physically active children tending to have lower quality of life [22]. A cluster randomized control trial, however, found that a physical activity intervention had no effect on HRQOL in preschool aged children in Canada [20]. The authors of the report stated that more research should be undertaken in preschool aged children, given that more physically active children are less likely to have difficulty walking [23], which is one measure of HRQOL.

The current study explores the association between objective accelerometer-measured physical activity with child HRQOL and family expenditure on physically activities. The Pediatric Quality of Life Inventory (PedsQL) [24] is an instrument that measures health-related quality of life in four domains: physical function, emotional function, social function and nursery function. To our knowledge, this present study is unique in comparing physical activity with the Pediatric Quality of Life Inventory (PedsQL) [24] as a measure of quality of life among 2–4-year olds in a UK community setting. We hypothesized that families that spent more on physical activity for their child would have a more active child and that children with higher PedsQL scores would be more physically active. However, the study remains exploratory, given that the trial was a feasibility study.

## Methods

The analyses were performed using baseline data from the Nutrition and Physical Activity Self-Assessment for Child Care UK (NAP SACC UK) feasibility randomized control trial [9]. NAP SACC UK is a nursery based intervention that employs elements of Social Cognitive Theory (SCT) within a socioecological framework [9]. The aim of NAP SACC UK is to improve the nutritional quality of food served, amount of physical activity and childcare settings’ policies around nutrition and physical activity. Further details on methods and study design are in the study protocol [9]. Baseline data collection was conducted in September to December 2015. Written informed consent was obtained by all children’s parents. Ethical approval for this study was given by Wales 3 NHS Research Ethics Committee.

### Participants

The study participants were recruited from 12 nurseries within two areas of south-west England: North Somerset and Gloucestershire. Children recruited were 2–4 years old and attended nurseries for at least 12 h per week.

### Description of measures

Study variables included physical activity, quality of life, expenditure on physical activity and demographic information.

*Physical activity* was measured using ActiGraph GT1M accelerometers which have been shown to provide accurate and reliable assessments of physical activity in children [25]. Accelerometers were worn for 7 days including weekends and days not in nursery. The accelerometers were set to record at 10s epochs [26]. To be included in the analysis the participants were required to have 2 days of valid data, either spent at nursery or not. Periods of ≥60 min with zero counts, allowing for 2 min of interruption, were taken as time the accelerometer was not worn and were removed from the analysis [25]. Parents and nursery staff were asked to remove the accelerometers during sleeping, bathing and swimming. A day was considered valid if 8 hours of data were recorded [27]. Data were processed to assess mean minutes of total physical activity (light, moderate and vigorous) and mean minutes of MVPA (moderate and vigorous physical activity). The thresholds for activity intensities were defined using criteria outlined by Puyau [28] as: sedentary (< 800 counts per minute (cpm)), light (LPA, 800 < 3200 cpm) and moderate-to-vigorous (MVPA, 3200 < 11,715 cpm) activity. A count value ≥11,715 cpm has been deemed “implausible” in previous literature and thus our data were capped at this value [29].

*Child’s quality of life* was measured using The Pediatric Quality of Life Inventory 4.0 (PedsQL) Generic Core Scales questionnaire for children aged 2–4 years [24]. Nursery staff distributed the questionnaires to parents along with a stamped addressed envelope to complete and return to the research team. The instrument has been tested for reliability and validity in community settings [24], including comparing preschool children with and without obesity [30]. The inventory has 21 items rating HRQOL in four domains: physical function, emotional function, social function and nursery function. Parents rated their child’s functioning over the past month related to each item on a scale of 0 to 4 (0 = never a problem, 1 = almost never a problem, 3 = often a problem, 4 = almost always a problem). The questionnaire with the PedsQL items can be found in Additional file 1. Items were reverse scored and linearly transformed onto a scale of 0–100, for ease of interpretation, so that higher PedsQL scores indicated better health-related quality of life. The physical health summary score is calculated as the mean score from the physical function scale (8 items). For the psychosocial summary score, the mean was computed as the sum of the items divided by the number of items answered in the emotional, social, and nursery function scales (15 items).

*Family expenditure on physical activity* of the 2–4-year old child was collected from a parent questionnaire. Parents were asked to list all the physical activities their 2–4-year old child had participated in over the past week and the associated cost of each activity (entry fees, membership etc.). A subsequent question asked how many miles in the car were travelled to get to the activity. Expenditure on transport was calculated using the HMRC tax rates per business mile, costed at 45p per mile [31]. The cost of transport to the activities was added to the expenditure on physical activities to generate the total weekly expenditure on physical activity of the 2–4-year-old.

*Demographic information* was collected via the parent questionnaire that was distributed by nursery staff. Parents were asked to give their highest educational attainment level, their child’s gender and child’s date of birth. Date of birth was converted into the child’s age and dichotomised into ‘2 years’ and ‘3–4 years’. The parental education options were: below GCSE/O-levels, up to GCSE/GCEs/O-levels or similar, A-levels/NVQs/GNVQs, first degree/diploma/HNC/HND, higher degree (e.g. MSc, PhD). GCSE/O-levels are compulsory qualifications taken in schools in England at age 16 years. A-levels/NVQs/GNVQs are optional qualifications taken in England in either sixth forms or further education colleges with A-levels being necessary to be accepted to UK universities. HNC/HND are higher education qualifications in the UK and can be equated to a university degree. MSc and PhD qualifications are post-graduate degrees. This parental education variable was dichotomised for analysis into ‘degree’ and ‘no degree’.

### Missing data

There was a high proportion of missing data in our sample (52%). A large proportion of accelerometer data was coded as missing as we worked to a threshold that required two full days of physical activity data. Open text questionnaires asking parents about money spent on physical activities additionally suffered from missingness. As this cross-sectional analysis uses data from a feasibility study, we did not find it appropriate to perform multiple imputation on exploratory analyses and instead report results from complete case analyses.

Missing PedsQL observations were imputed, as per the PedsQL scoring instructions [32], as there were no participants that had > 50% of scores missing from any one function scale.

### Statistical analysis

Medians and inter-quartile ranges of physical activity expenditure, and PedsQL physical and psychosocial scores were presented for gender, age, parental education groups, with null hypotheses of no difference in medians between groups being tested with the Mann-Whitney U test. Means and standard deviations of minutes in physical activity and MVPA were presented for gender, age, parental education, and nursery / non-nursery day groups, with the null hypotheses of no difference in means between groups being tested with the Student t-test. Separate mixed effects linear regression models evaluated the strength of associations between measures of physical activity as the outcome variables (mean minutes in active time and mean minutes in MVPA), and PedsQL scores and the family expenditure on physical activity as covariates. Variation in mean activity between nurseries was accommodated by including nursery as a random effect. We adjusted models for age, gender and parental education as possible confounders. Expenditure on physical activity and the PedsQL psychosocial summary scores were each included as four ordered categories, as both variables were heavily skewed. PedsQL physical functioning scores were analysed in tertiles due to more than 25% of the sample having the maximum score of 100. Evidence against the null hypothesis of no trend in mean activity measure across ordered categories of parental expenditure and PedsQL was evaluated using the test for trend. All analyses were conducted in Stata Version 14.2 (StataCorp, College Station, Texas, USA, 2015).

## Results

**Fig. 1:**
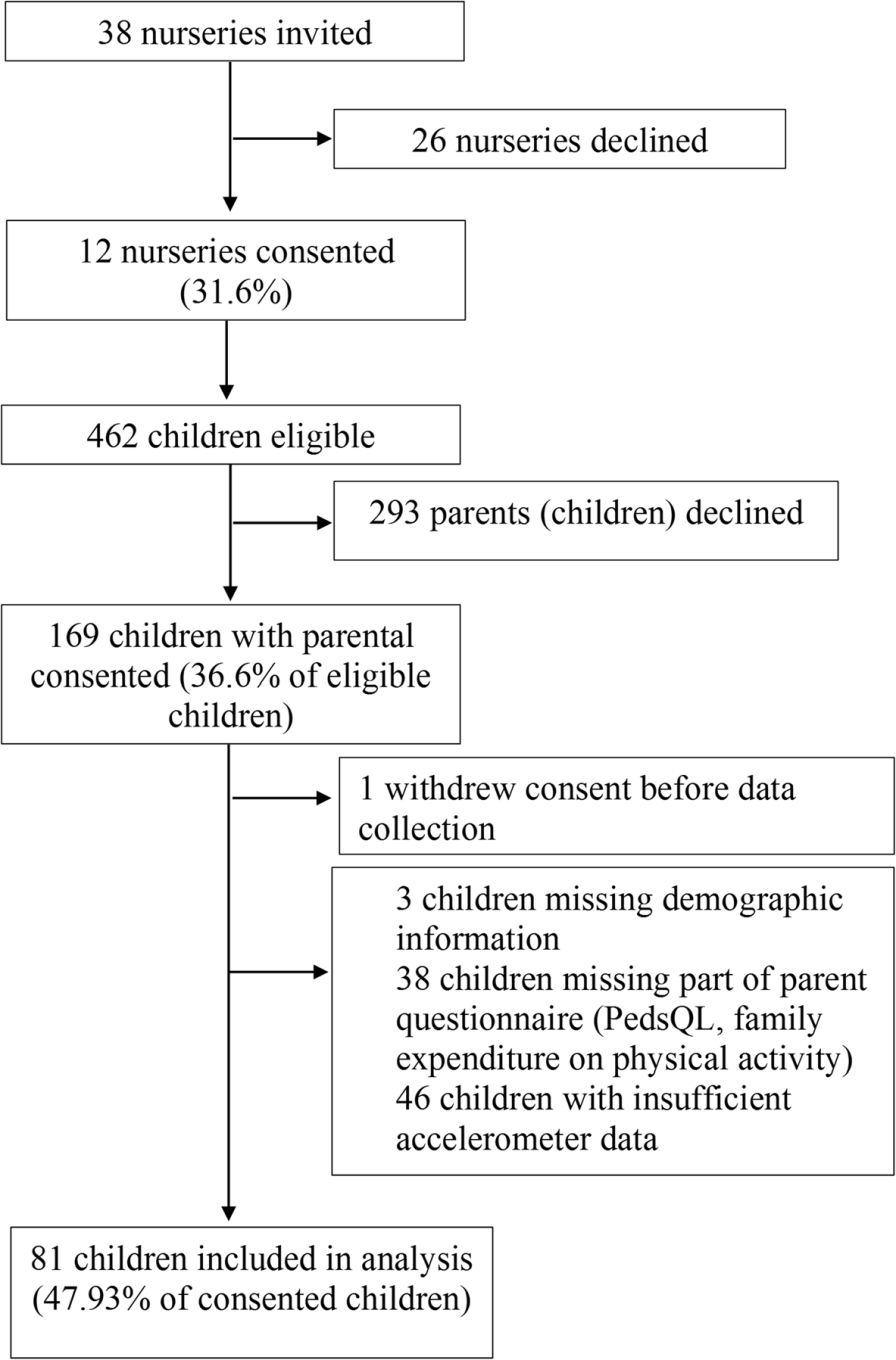
Flow diagram of participants included in the sample. The diagram shows the derivation of the sample in this study. It shows participants recruited into the NAP SACC UK trial and describes where participants withdrew or there was missing data, resulting in the final sample

There were 169 children who had consent for data collection at baseline. Figure 1 shows that the final sample that had complete data for all the measures was 81 children. The majority of the children in the complete data sample had a parent with a university degree (74%). There was 22.2% of the sample with a parent whose highest educational attainment was A-Levels. There was no statistical difference in parental education between the children included in the sample and those for which we had consent but were not included in the sample.

In this sample, with data collected in the autumn, 88.9% of children did not meet the recommended 180 min of daily physical activity. Table 1 presents the means (SD) of minutes in daily physical activity and minutes in daily MVPA. Participating children (*n* = 81) had a mean (SD) of 141.9 (33.1) minutes of daily physical activity and a mean (SD) of 22.2 (9.9) minutes of daily MVPA. Children spent more minutes being physically active on nursery days compared with non-nursery days (146.9 vs. 137.2, *p* = 0.05). There was no evidence of difference in minutes spent in MVPA on nursery days compared to non-nursery days (22.8 vs. 21.7, *p* = 0.4).

**Table 1:** Time spent physically active and in MVPA by gender, age, parent education and nursery/non-nursery day

	Physical activity (light, moderate and vigorous) (minutes per day)		MVPA (minutes per day)	
	Mean (SD)	*p*-value^1^	Mean (SD)	*p*-value^4^
Whole sample (*n* = 81)	141.9 (33.1)	*–*	22.2 (9.9)	–
Gender				
Male (*n* = 43)	148.9 (33.5)		24.50 (11.0)	
Female (*n* = 38)	133.9 (31.3)	0.04	19.61 (7.7)	0.03
Age^2^				
2 years old (*n* = 38)	131.2 (31.1)		18.29 (8.1)	
3–4 years old (*n* = 43)	151.3 (32.3)	0.01	25.67 (10.1)	0.0006
Parent Education				
Degree (*n* = 59)	140.3 (32.1)		21.65 (9.2)	
No degree (*n* = 22)	146.2 (36.1)	0.48	23.70 (11.5)	0.41
Nursery day/non-nursery day (*n* = 78)^3^				
Nursery day	146.9 (44.3)		22.76 (13.3)	
Non-nursery day	137.2 (33.8)	0.05	21.67 (10.4)	0.40

^1^ *p*-value for the mean difference based on t-test

^2^ 10 children aged 4-years at time of consent

^3^ *n* = 78 as some children did not have enough data for either nursery or non-nursery days

Boys were more physically active than girls, spending 149.0 vs. 133.9 min in daily activity (*p* = 0.04). Boys also spent more time in MVPA than girls (24.5 vs. 19.6 min, p = 0.05). There were also mean differences between the age groups, with older children being more physically active (*p* = 0.01) and spending more time in MVPA (*p* = 0.005) than younger children. There was no evidence of differences for any of the physical activity variables by parental education.

Table 2 presents the baseline medians and interquartile range (IQR) of weekly expenditure on physical activity, PedsQL physical and psychosocial summary scores. The median (IQR) for family expenditure on weekly physical activity of the 2–4-year-old child was £13.37 (£3.00–16.37). Further, 50.6% of the sample spent less than £9.00 weekly on their 2–4-year-old’s physical activity, with 17.3% spending no money on physical activities. PedsQL scores for the sample were skewed. For the physical functioning summary score, the median (IQR) was 93.8 (84.4–100). The psychosocial summary score was lower than the physical score, with a median (IQR) of 86.5 (80.8–92.3). There was no evidence of difference in scores by gender, age or parental education.

**Table 2:** Baseline medians for physical activity and PedsQL scores by gender, age and parent education^1^

	Weekly expenditure on physical activity for 1–4 year old (£)		PedsQL Physical Functioning (scale 0–100)		PedsQL Psychosocial Functioning (scale 0–100)	
	Median (IQR)	*p*-value^2^	Median (IQR)	*p*-value^2^	Median (IQR)	*p*-value^2^
Whole sample (*n* = 81)	13.4 (3.0–16.4)	–	93.8 (84.3–100)	–	86.5 (80.8–92.3)	–
Gender						
Male (*n* = 43)	9.4 (0.9–18.4)		93.8 (87.5–100)		86.5 (80.8–94.2)	
Female (*n* = 38)	6.4 (3.0–14.8)	0.56	90.6 (82.3–96.88)	0.27	86.5 (78.9–92.3)	0.70
Age						
2 years old (*n* = 38)	9.0 (1.5–16.8)		92.2 (84.4–100)		86.5 (78.9–92.3)	
3–4 years old (*n* = 43)^3^	7.2 (4.5–16.4)	0.62	93.8 (87.5–100)	0.60	88.5 (82.7–92.3)	0.27
Parent Education						
Degree (*n* = 59)	6.8 (3.3–15.7)		93.8 (87.5–100)		88.5 (80.8–92.3)	
No degree (*n* = 22)	10.0 (2.3–17.3)	0.59	93.8 (81.3–100)	0.85	82.7 (78.8–90.4)	0.15

^1^ Table testing two study hypotheses by fitting mixed-effects linear regression models

^2^ *p*-value for the mean difference based on t-test

^3^ Ten children aged 4 years

Table 3 shows that for physical activity (light, moderate and vigorous) Tertile 3 is associated with 5.2 more minutes of physical activity than the reference group (95% CI -12.1, 22.4), with confidence intervals crossing the null. The trend test *p*-value showed no evidence for a trend across the physical functioning tertiles (*p* = 0.58).

**Table 3:** Mixed-effects regression of physical activity, PedsQL and expenditure on physical activity (£) of 2–4-year-old children

Adjusted models^1^	Physical activity (Light, moderate and vigorous)				MVPA (Moderate and vigorous)			
	Coefficient	95% CI	*p*-value^2^	Trend test^3^	Coefficient	95% CI	*p*-value^2^	Trend test^3^
Covariate								
PedsQL Physical Functioning score (scale 0–100)								
Reference group: 50.00–87.50	–	–	–		–	–	–	
Tertile 2: 87.51–93.75	5.1	−11.9, 22.1	0.56		3.32	−1.5, 8.1	0.18	
Tertile 3: 93.76–100	5.2	−12.1, 22.4	0.56	0.58	3.6	−1.3, 8.4	0.15	0.18
PedsQL Psychosocial Functioning score (scale 0–100)								
Reference group: 51.92–80.77	–	–	–		–	–	–	
Quartile 2: 80.68–86.54	10.7	−7.1, 28.6	0.24		2.2	−2.9, 7.3	0.40	
Quartile 3: 86.55–92.31	14.5	−3.2, 32.2	0.11		5.8	−0.8, 10.8	0.02	
Quartile 4: 92.32–100	−1.5	−19.4, 16.4	0.87	0.88	1.2	−3.9, 6.3	0.48	0.32
Total weekly expenditure on physical activity (£)^4^								
Reference group: 0.00–2.25	–	–	–		–	–	–	
Quartile 2: 2.26–7.20	−10.1	−29.9, 9.7	0.32		0.7	−4.9, 6.3	0.81	
Quartile 3: 7.21–16.70	−1.8	−21.9, 18.1	0.86		−0.2	−5.8, 5.5	0.95	
Quartile 4: 16.71–47.15	2.1	−17.9, 22.1	0.84	0.68	0.2	−5.4, 5.9	0.93	0.996

^1^ Adjusted for parental education, child age, gender and cluster

^2^ *p*-values for difference between each tertile/quartile and the reference group

^3^ *p*-value for trend test across tertiles or quartiles

^4^ Minutes on non-nursery days were used under the assumption that spending on physical activity for the 2–4-year-old would occur on non-nursery days

There no evidence (Table 3) of an association between PedsQL physical functioning and mean minutes in daily MVPA. Tertile 2 (scores ranging from 87.51–93.75) was associated with 3.3 more minutes in MVPA than the reference group with the lowest physical functioning scores (95% CI: − 1.5, 8.1). Tertile 3 (scores ranging from 93.76–100) was associated with 3.6 min more MVPA than the reference group (95% CI: − 1.3, 8.4). The trend test p-value (*p* = 0.18) showed no evidence for a trend across the physical functioning tertiles. The unadjusted model (Appendix 1) showed very weak evidence for a trend across the physical functioning tertiles (*p* = 0.06), that attenuated after adjusting for confounding variables.

For the PedsQL psychosocial summary score, the regression models for physical activity (Table 3) show that Quartile 3 was associated with 14.5 min more active time than the reference group (95% CI: − 3.2, 32.2). For MVPA, the quartile with the highest PedsQL psychosocial scores (Quartile 4) was associated with 1.2 more minutes in MVPA compared with the reference group (95% CI: − 3.9, 6.3). Quartile 3, with psychosocial scores ranging from 86.55–92.31, was associated with 5.8 min more time in MVPA (95% CI: − 0.8, 10.8) than the reference group. The trend test *p*-value showed no evidence of a trend across the psychosocial quartiles for physical activity (*p* = 0.88) or MVPA (*p* = 0.32).

For the physical activity expenditure variable, the regression model in Table 3 shows no evidence of a relationship between the amount of money spent on the 2–4-year-old being physically active and minutes in daily activity or daily MVPA. However, the differences in mean activity indicate that most quartiles engaged in less physical activity and time in MVPA compared with the reference group, which contains the lowest family expenditure (£0–2.25 weekly) on the child’s physical activity.

## Discussion

This study found that 88.9% of 2–4-year-old children are not meeting the recommended 180 min of daily physical activity. This replicates results from previous studies [2, 4]. Boys were more physically active than girls, which is consistent with previous studies showing boys engage in more and higher intensity physical activity than girls at pre-school age [33–36]. Children aged 3–4 years were more physically active and spent more time in MVPA than children aged 1–2 years, which would be expected due to older children being more developmentally capable of more frequent and higher intensity movement [2]. Children in this sample are more physically active on nursery days than non-nursery days. This study adds to the literature by highlighting low physical activity levels in preschool aged children, supporting the case for health promotion interventions in this age group. It additionally highlights the need to work with parents and carers to promote physical activity outside nursery.

There was no difference in HRQOL by gender, age of child or parental education (Table 2), This finding, however, is not unexpected, as HRQOL differences have been found between obese and overweight pre-schoolers [22] and migrant and non-migrant pre-schoolers [18], but there is no evidence of a difference between 2 and 4-year-olds in UK community populations. It may be the case that young children in healthy, community settings a relatively similar in terms of their HRQOL. However, the small sample size means these findings are tentative.

There was no evidence of an association between PedsQL physical functioning (such as walking, running and lifting things) scores and time spent in MVPA (Table 3). There was very weak evidence of an association between these variables in the unadjusted model (Table 4). However, the confidence intervals crossed the null, meaning this finding could be due to chance. Table 3 also shows no evidence of an association between and psychosocial functioning scores and physical activity (light, moderate and vigorous) or MVPA, with no evidence of trend across the quartiles or tertiles. Therefore, the first hypothesis was rejected. Taking the association between psychosocial functioning and physical activity and MVPA as an example, the coefficients for Quartile 2 and Quartile 3 are much greater for physical activity than MVPA (Quartile 2: 10.7 vs 2.2, Quartile 3: 14.5 vs 5.8). Indicatively, this means that PedsQL has a greater effect on light physical activity than on moderate-to-vigorous physical activity. Future research with larger samples should interrogate this further, as any observed effect on total physical activity could be mostly attributably to light physical activity. Given the benefits of MVPA, it is important to make this distinction. However, the lack of power in this present study means these findings are only illustrative.

These findings contrast previous research that found associations between physical activity and HRQOL in other age groups [22]. The age of the participants may have impacted upon these results, as HRQOL is reported by the parents on behalf of the child. Previous work has shown parents are able to rate some expects of HRQOL better than others [37]. However, the lack of association between physical activity and HRQOL mirrors Truelove et al’s [20] experimental study, which found that a physical activity intervention had no impact on pre-schoolers’ PedsQL scores. Further research with larger samples is required to test whether the null finding in our study is due to sample size.

We found no evidence of an association between parental expenditure on physical activities and minutes the child spent in physical activity or MVPA, thus our second hypothesis was not supported. This null finding is in contrast to previous work that has found cost to be an important barrier to physical activity participation [14]. There are a number of possible reasons for the lack of association in this sample. Firstly, it may be the case that some parents spend more money on physical activities for their child, because their child exhibits low levels of physical activity and so they hope to encourage them to do more. Secondly, for some children there are opportunities for free physical activities, such as playing in the garden or in the park. Therefore, access to such spaces may be a more important factor than actual funds spent on activities. Previous research has found increased proximity between homes and park areas to be associated with higher levels of physical activity in young children [38]. A large proportion of the sample spent nothing on physical activities. Further, the small sample size as well as the high proportion of parents from high socioeconomic backgrounds, 74% compared to 40.3% of adults aged 25–34 and 33.6% of adults aged 35–49 in England and Wales [39], could have resulted in the lack of association between physical activity and expenditure on physical activities.

### Strengths and limitations

This study is among the first to employ PedsQL as a quality of life measure in this age group, and the first to our knowledge in the UK to use PedsQL among children in a community setting and assess links with physical activity. The study, does however, have limitations. This study was cross-sectional; thus, it cannot be used to determine causal effects of expenditure and PedsQL scores on physical activity levels. As a feasibility trial, the sample size was small, and we had limited statistical power to test for differences in some outcomes we hypothesized. However, we had power to detect a five-minute difference in MVPA between genders. Issues with the amount of usable days of accelerometer data from the children meant that the sample size was further reduced. The *p*-values testing trend across the categorical variables were also high. A further limitation to the study is that the sample is disproportionately made up of children whose parents are of high socioeconomic status based on their education. Although nurseries were recruited from areas of varying neighbourhood deprivation scores, parents in the sample have relatively high education compared to the general population. These parents are likely to be more affluent and have more disposable income to spend on physical activity. Overall, the sample cannot be said to be representative.

## Conclusions

This exploratory study demonstrates 88.9% children aged 2–4 years old fail to meet physical activity guidelines. There were higher levels of physical activity on nursery days compared with non-nursery days. Our hypothesis that HRQOL and physical activity are associated in young children was not supported by these data. There appears to be no evidence of an association between money spent on physical activity and levels of physical activity or MVPA. This should be explored further, as the lack of association potentially conflicts with the hypothesis of cost as a barrier to physical activity participation. However, for this more affluent sample, this hypothesis may not be relevant within this context as there are not many high cost activities for young children. Replication in larger, more representative samples is required.

### Additional file

Additional file 1: PedsQL parent questionnaire**.** The questionnaire given to parents to complete on behalf of their 2–4-year-old child. Parents gave their child a score of 0–5 based on a series of physical and psychosocial parameters such as walking, running, feeling scared, playing with other children. A score of 0 indicated the child never has problems with the parameter and 5 indicated they always have problems. (DOC 49 kb)

## Data Availability

The datasets used and/or analysed during the current study are available from the corresponding author on reasonable request.

## Appendix

### Unadjusted models

**Table 4:** Mixed-effects regression of physical activity, PedsQL and expenditure on physical activity (£) of 2–4-year-old children

Unadjusted models	Physical activity (Light, moderate and vigorous)				MVPA (Moderate and vigorous)			
	Coefficient	95% CI	*p*-value^1^	Trend test^2^	Coefficient	95% CI	*p*-value^2^	Trend test^a^
Covariate								
PedsQL Physical Functioning score (scale 0–100)								
Reference group: 50.00–87.50	–	–	–		–	–	–	
Tertile 2: 87.51–93.75	11.0	−8.0, 28.4	0.27		5.7	0.4, 11.0	0.04	
Tertile 3: 93.76–100	8.8	−7.7, 25.3	0.29	0.29	4.5	−0.1, 9.5	0.06	0.06
PedsQL Psychosocial Functioning score (scale 0–100)								
Reference group: 51.92–80.77	–	–	–		–	–	–	
Quartile 2: 80.68–86.54	13.2	−6.6, 32.9	0.19		3.3	−2.6, 9.1	0.28	
Quartile 3: 86.55–92.31	12.3	− 6.6, 31.3	0.20		5.0	−.6, 10.7	0.08	
Quartile 4: 92.32–100	1.6	−18.2, 21.3	0.88	0.76	2.4	−3.5, 8.2	0.43	0.28
Total weekly expenditure on physical activity (£)^3^								
Reference group: 0.00–2.25	–	–	–		–	–	–	
Quartile 2: 2.26–7.20	−12.1	−33.2, 9.0	0.26		0.3	−6.1, 6.7	0.94	
Quartile 3: 7.21–16.70	− 6.0	−26.6, 14.6	0.57		−1.0	−7.2, 5.2	0.75	
Quartile 4: 16.71–47.15	−0.5	−21.8, 20.9	0.96	0.91	−0.2	−6.6, 6.2	0.95	0.85

^1^ *p*-values for difference between each tertile/quartile and the reference group

^2^ *p*-value for trend test across tertiles or quartiles

^a^Minutes on non-nursery days were used under the assumption that spending on physical activity for the 2–4-year-old would occur on non-nursery days

## Abbreviations

A-level: Advanced Level
CI: Confidence Interval
CPM: Counts per minute
GCSE: General Certificate of Secondary Education
GNVQ: General National Vocational Qualification
HMRC: Her Majesty’s Revenue and Customs
HRQOL: Health Related Quality of Life
IQR: Interquartile range
LPA: Light physical activity
MSc: Master of Science
MVPA: Moderate-vigorous physical activity
N: Number
NAP SACC UK: Nutrition and Physical Activity Self-Assessment for Child Care UK
NHS: National Health Service
NVQ: National Vocational Qualification
O-Level: Ordinary Level
p: Pence
PedsQL: Pediatric Quality of Life Inventory
PhD: Doctor of Philosophy
SD: Standard Deviation
WHO: World Health Organisation
