# Supplementary material for: Cross-sectional analysis of physical activity in 2–4-year-olds in England with paediatric quality of life and family expenditure on physical activity
Source: BMC Public Health. 2019 Jun 28;19:846. doi: 10.1186/s12889-019-7129-y (PMC6599301; doi:10.1186/s12889-019-7129-y)
Supplement: Supplementary file 1 — PedsQL parent questionnaire. The questionnaire given to parents to complete on behalf of their 2–4-year-old child. Parents gave their child a score of 0–5 based on a series of physical and psychosocial parameters such as walking, running, feeling scared, playing with other children. A score of 0 indicated the child never has problems with the parameter and 5 indicated they always have problems. (DOC 49 kb) [file 12889_2019_7129_MOESM1_ESM.doc]

*In the past* ***ONE month,*** *how much of a* ***problem*** *has your child had with …*

| Physical Functioning *(problems with…)* | Never | **Almost Never** | Some-times | **Often** | Almost Always |
| --- | --- | --- | --- | --- | --- |
| 1. Walking | 0 | 1 | 2 | 3 | 4 |
| 2. Running | 0 | 1 | 2 | 3 | 4 |
| 3. Participating in sports or running games | 0 | 1 | 2 | 3 | 4 |
| 4. Lifting heavy things | 0 | 1 | 2 | 3 | 4 |
| 5. Bathing | 0 | 1 | 2 | 3 | 4 |
| 6. Helping to pick up his or her toys | 0 | 1 | 2 | 3 | 4 |
| 7. Having hurts or aches | 0 | 1 | 2 | 3 | 4 |
| 8. Feeling very tired | 0 | 1 | 2 | 3 | 4 |

| Emotional Functioning *(problems with…)* | Never | **Almost Never** | Some-times | **Often** | Almost Always |
| --- | --- | --- | --- | --- | --- |
| 1. Feeling afraid or scared | 0 | 1 | 2 | 3 | 4 |
| 2. Feeling sad or unhappy | 0 | 1 | 2 | 3 | 4 |
| 3. Feeling angry or cross | 0 | 1 | 2 | 3 | 4 |
| 4. Trouble sleeping at night | 0 | 1 | 2 | 3 | 4 |
| 5. Worrying about what will happen to him or her | 0 | 1 | 2 | 3 | 4 |

| Social Functioning *(problems with…)* | Never | **Almost Never** | Some-times | **Often** | Almost Always |
| --- | --- | --- | --- | --- | --- |
| 1. Playing with other children | 0 | 1 | 2 | 3 | 4 |
| 2. Other kids not wanting to play with him or her | 0 | 1 | 2 | 3 | 4 |
| 3. Getting teased by other children | 0 | 1 | 2 | 3 | 4 |
| 4. Not able to do things that other children his or her  age can do | 0 | 1 | 2 | 3 | 4 |
| 5. Keeping up when playing with other children | 0 | 1 | 2 | 3 | 4 |

| Nursery/School Functioning *(problems with…)* | Never | **Almost Never** | Some-times | **Often** | Almost Always |
| --- | --- | --- | --- | --- | --- |
| 1. Doing the same activities as peers | 0 | 1 | 2 | 3 | 4 |
| 2. Missing nursery/school because of feeling ill  well | 0 | 1 | 2 | 3 | 4 |
| 3. Having days off nursery/school to go to the doctor or hospital | 0 | 1 | 2 | 3 | 4 |
